# Platelets as delivery vehicles for targeted enrichment of NO^·^ to cerebral glioma for magnetic resonance imaging

**DOI:** 10.1186/s12951-023-02245-y

**Published:** 2023-12-21

**Authors:** Yuchen Ding, Min Ge, Chao Zhang, Juncheng Yu, Donglin Xia, Jian He, Zhongzheng Jia

**Affiliations:** 1 Department of Medical Imaging, Affiliated Hospital of Nantong University, School of Public Health of Nantong University, Medical School of Nantong University, Nantong, 226001 PR China; 2 Institute of Biology and Nanotechnology of Nantong University, Nantong, 226019 PR China; 3 Department of Nuclear Medicine, Nanjing Drum Tower Hospital, Affiliated Hospital of Medical School, Nanjing University, Nanjing, Jiangsu 210008 PR China; 4 Department of Neurosurgery Center, Zhujiang Hospital, Southern Medical University, Guangzhou, 510282 PR China

**Keywords:** Platelets, Nano free radical nitric oxide micelles, Magnetic resonance imaging, Glioma, Target

## Abstract

Using a magnetic resonance imaging (MRI) contrast agent, MRI has made substantial contributions to glioma diagnosis. Metal-free MRI agents, such as the nano free radical nitric oxide (NO^·^) micelle, can overcome the inherent toxicity of metal-based agents in certain patient populations. However, the low spatial resolution of nano NO^·^ micelle in MRI limits its clinical development. In this study, we pretreated platelets (PLTs) and loaded them with nano NO^·^ micelles to synthesize NO^·^@PLT, which can overcome the low contrast and poor in vivo stability of nitroxide-based MRI contrast agents. The PLTs can serve as potential drug carriers for targeting and delivering nano NO^·^ micelles to gliomas and thus increase the contrast in T1-weighted imaging (T1WI) of MRI. This drug carrier system uses the unique tumor-targeting ability of PLTs and takes advantage of the high signal presentation of steady nano NO^·^ micelles in T1WI, thereby ultimately achieving signal amplification of glioma in T1WI. With the effect of PLTs-tumor cell adhesion, NO^·^@PLT has per-nitroxide transverse relativities of approximately 2-fold greater than those of free NO^·^ particles. These features allow a sufficient NO^·^@PLT concentration to accumulate in murine subcutaneous glioma tumors up from 5 min to 2.5 h (optimum at 1.5 h) after systemic administration. This results in MRI contrast comparable to that of metal-based agents. This study established a promising metal-free MRI contrast agent, NO^·^@PLT, for glioma diagnosis, because it has superior spatial resolution owing to its high glioma-targeting ability and has significant translational implications in the clinic.

**Supplementary Information:**

The online version contains supplementary material available at 10.1186/s12951-023-02245-y.

## Introduction

Due to its extremely invasive nature, poor prognosis, and high fatality rate, glioma is an intracranial malignant tumor that presents a significant clinical challenge [1]. Magnetic resonance imaging (MRI), a noninvasive imaging modality, is a crucial player in glioma diagnosis [2–4]. MRI can help visualize the boundary between the tumor and normal tissue and thus make a diagnosis about glioma subtypes [5, 6]. This is because MRI has a high spatial resolution and can penetrate tissue, but it still has drawbacks like limited specificity and sensitivity [7, 8]. Moreover, contrast agents are often required to improve the diagnostic accuracy of MRI in gliomas, and metal-containing contrast agents are commonly used [9–13]. Gadolinium contrast agents, which reduce T1 relaxation time to improve image contrast and highlight more tumor characteristics in MRI scans, are frequently utilized in clinical settings [14–16]. For T1-weighted and T2-weighted images, the signals correspond to spin-lattice relaxation and spin-spin relaxation, respectively. Although there are cutting-edge molecular imaging approaches for the diagnosis of gliomas, their usage in real-time intraoperative imaging is constrained due to their poor spatial resolution and potential heavy metal toxicity [17–19]. Thus, there is growing interest in creating “metal-free” MRI contrast agents made solely of organic materials [20].

One of the metal-free MRI contrast agents that has drawn a lot of research attention is nitroxide-based organic radical contrast agents [21, 22]. These nitroxide-based contrast agents, as organic substances, can theoretically reduce the toxicity affecting organisms, have better biocompatibility [20, 23], and create MRI contrast by using conventional water relaxation methods. In theory, they may be applied right away in therapeutic settings. The therapeutic use of these nitroxide-based contrast agents is nevertheless constrained by a number of significant problems.

First, there is just one unpaired electron present in nitroxide radicals. Consequently, compared with metal-based contrast agents such as Gd^3+^ (seven unpaired electrons) or Mn^2+^ (five unpaired electrons), nitroxide-based organic radical contrast agents inherently suffer from considerably lower water ^1^H relaxivity [24]. Furthermore, nitroxide-based organic radical contrast agents are easily captured by phagocytes or reticuloendothelial system in internal circulation and their aggregation and imaging at the tumor are difficult [25–28]. Additionally, they frequently transform quickly into diamagnetic hydroxyl amines in vivo (half-lives on the order of minutes), which makes them useless as contrast agents soon after injection. These shortcomings were evident while making initial efforts to utilize nitroxides as MRI contrast agents. The development of in vivo-stable nitroxide-based organic radical contrast agents that enable longitudinal studies over clinically significant time scales after systemic administration has not yet occurred, despite cleverly utilizing their rapid bioreduction to enable redox mapping in vitro and in vivo.

One strategy for achieving higher molecular relativity rate is to accumulate free radical nitric oxide (NO^·^) at tumor sites through targeting strategies. Thus far, numerous methods for targeted distribution have so far been created and even used in medicine, including the utilization of blood cells [29]. Platelets (PLTs) have served as an inspiration for the creation of drug targeted carriers. The circulation half-life of PLTs is about 30 h. Part of the reason for this prolonged circulation can be attributed to CD47, a “marker-of-self” found on PLTs that interacts with immune cells’ signal regulatory protein (SIRP) and prevents immune clearance [30].

Tumor-targeted drug delivery is highly desirable for tumor diagnosis or treatment, and PLTs have emerged as intriguing candidate drug carriers to meet this need. For protection, P-selectin, a cubicle adhesion scintilla upregulated in activated PLTs, ass group to P-selectin glycoprotein ligand-1 (PSGL-1) or CD44 overexpressed on tumor cells, thereby allowing complex and dynamic PLT–tumor cross-talk critical for tumor growth and metastasis. We also exploited the stimulus responsiveness and modifiability of PLTs to construct a multifunctional drug delivery vector [31–33]. PLT drug delivery systems inherit these dynamic binding properties from the source PLTs for active drug targeting. Such broad and dynamic biointerfacing capabilities have made PLTs attractive drug carriers for targeted delivery applications [32].

**Scheme 1:**
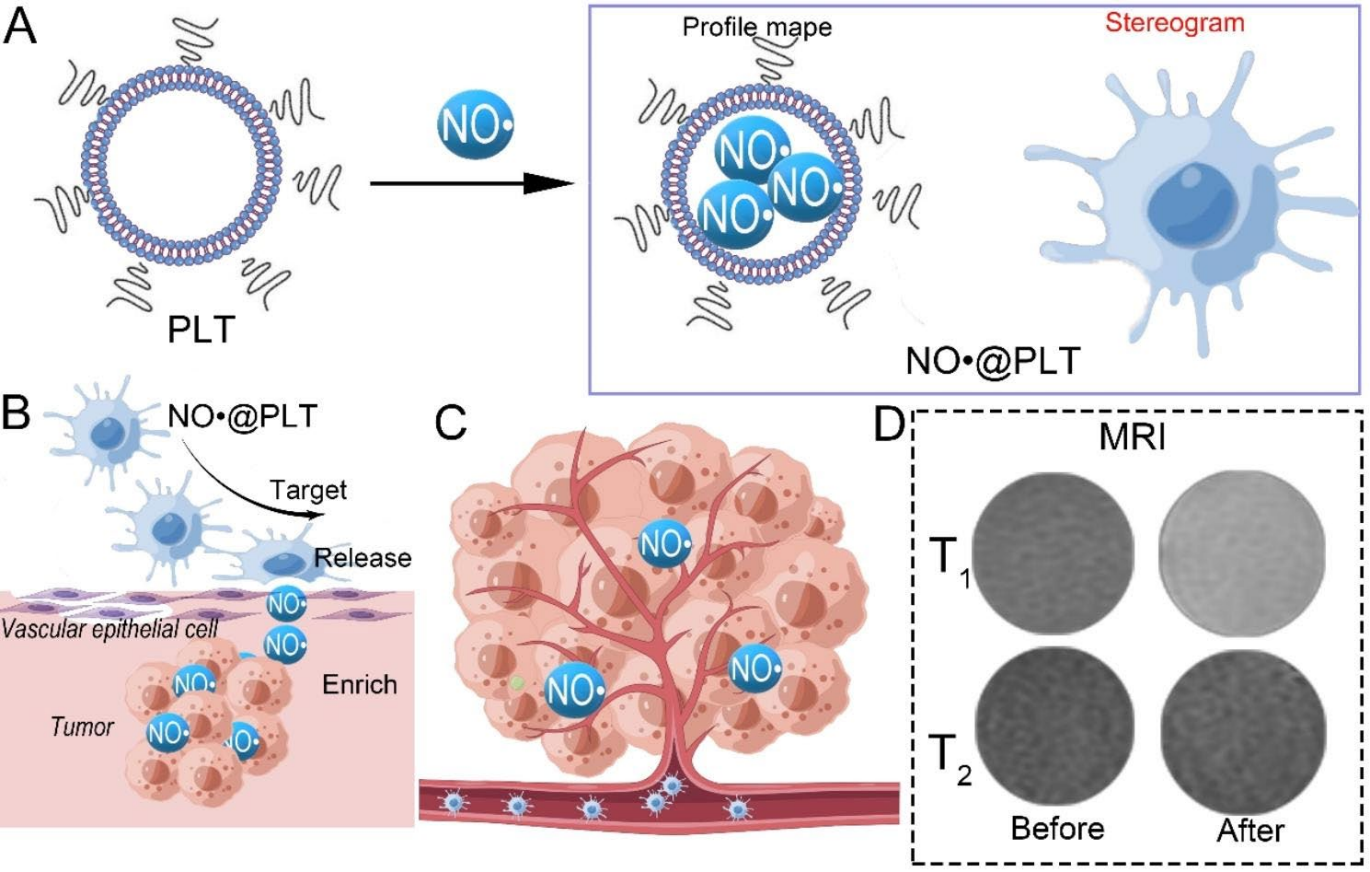
Schematic illustration of platelets as delivery vehicles for NO^·^ (NO^·^@PLT) for magnetic resonance imaging. (**A**) Schematic illustration depicting the synthesis of NO^·^@PLT. (**B**) NO^·^@PLT targeted the tumor and released NO^·^ micelles. (**C**) Nano NO^·^ micelles were enriched in the cerebral glioma. (**D**) The T1-weighted MRI phantoms were increased after NO^·^@PLT treatment

Here, we outline a strategy for the diagnosis of glioma through targeted delivery of adequate NO^·^ to the glioma for MRI (Scheme 1). In brief, the metal-free MRI contrast agent (nano NO^·^ micelles) was entrapped within the PLTs to form the NO^·^ loaded PLT particles (NO^·^@PLT) (Scheme 1A). This contrast agent recognizes receptors on glioma cells through the membrane proteins of PLTs and finally targets the tumor. Then, the oxygen-depleted environment of high ADP in the tumor causes PLT aggregation and activation, thereby releasing NO^·^ and realizing the high signal of tumors in MRI (Scheme 1B). We expected that these modified PLTs could maximize the drug-carrying ability and enhance NO^·^ release, so as to enrich the tumor with NO^·^ (Scheme 1C), increase the efficiency of early glioma diagnosis and improve the prognosis of tumor patients (Scheme 1D).

## Materials and methods

### Materials and animals

4-Amino-2,2,6,6-tetramethylpiperidine (NO^·^), sulfo-cyanine5 (Cy5), glutamic acid and prostaglandin E1(PGE1) were purchased from Bomei Biotechnology (Hefei, China). CD41 antibody and immunoglobulin G (IgG) were purchased from Bioss Biotechnology (Beijing, China). A fluorescein isothiocyanate isomer (FITC) was obtained from Zhongke Chenyu Biotechnology (Beijing, China). The CCK8 assay was performed using Cell Counting Kit-8 (CCK8, Biyuntian, Haimen, China). 1-Ethyl-3-(3-dimethylaminopropyl) carbodiimide (EDC) and N-hydroxysuccinimide (NHS) were obtained from Sigma-Aldrich (Munich, Germany). All reagents used were of analytical grade.

The Human Anatomy Laboratory of Nantong University provided U87 cells and HULEC-5a epithelial cells. The cells were cultured at 37 °C under 5% CO_2_ in 10% fetal bovine serum, 100 U/mL penicillin G and 100 mg/mL streptomycin. Male BALB/c mice (weight: 15 ± 2 g) and Sprague–Dawley (SD) rats (weight: approximately 150 g) were purchased from the Laboratory Animal Center of Nantong University. The animals were housed at 24 ± 2 °C, 40–70% humidity, and a 12-h photoperiod. All experimental procedures were approved under protocol number S20221222-007 by the Director of IACUC of Nantong University.

### Synthesis of NO^·^@PLT

The core-shell type nano NO^·^ micelles were constructed as follows: Acetal-PEG-SH was prepared as Akiyama described [34]. The obtained acetal-PEG-b-PCMS was mixed slowly with an excess solution of 4-amino-2,2,6,6-tetramethylpiperidine (pH 10) and incubated for 20 min at room temperature. The reacted polymer was recovered by precipitation into cold 2-propano and centrifugated at 5000 rpm. The precipitation-centrifugation cycle was preformed to purify the obtained polymer. To complete the process, the obtained polymer was dissolved in DMF, transferred into a membrane tube preswollen with a molecular weight cutoff of 3500, and dialyzed for 24 h.

Fresh blood collected from the SD rats was injected into a heparin-infiltrated anticoagulation tube. The blood was then transferred to a centrifuge tube and centrifuged twice at 200 rpm for 10 min to obtain plasma. Then, PGE1 was added to the solution to improve the ability of PLTs as a drug carrier. The supernatant was collected and centrifuged at 1800 ×*g* for 20 min. A predetermined amount of NO^·^ (10 mg/mL) was added to the extracted PLTs. The permeability of the PLT membrane was changed through ultrasonic treatment (80 W, 40 Hz) at ambient temperature, thereby leading to the preparation of NO^·^@PLT.

### Characterization of NO^·^@PLT

Scanning electron microscopy (SEM) was used to characterize morphological changes during NO^·^@PLT preparation. First, 2.5% (v/v) glutaraldehyde was added to PLTs and NO^·^@PLT to fix their form at 4 °C for 24 h. The samples were then centrifuged (room temperature, 1800 ×*g* for 10 min) to discard the supernatant, and 2 mL PBS (pH 7.2) was added to dilute the solution. The suspension was dropped onto a silicon wafer and dried in a baking oven at 70 °C for 30 min. The dried samples were examined under a JSM-6700 F microscope (JEOL, Japan). Zeta sizer Nano (ZS90, Malvern Instruments, England) was used to measure the zeta potential and particle size of nano NO^·^ micelles, NO^·^@PLT, and PLTs. The signal of nano NO^·^ micelles in the NO^·^@PLT was tested by conducting electron paramagnetic resonance (EPR) experiments at room temperature by using a Bruker EMXplus-10/12 spectrometer (Bruker, Germany). The NO^·^ concentration of each sample was also measured by X-band EPR. All analyses were performed in triplicate.

To construct the FITC-labeled NO^·^@PLT, the FITC were added into the obtained acetal-PEG-b-PCMS-NO^·^. Afterward, the mixture was dialyzed in deionized water for 12 h to remove the free FITC, resulting in FITC-labeled nano NO^·^ micelles. Then, the FITC labeled nano NO^·^ micelles were used in the construction of NO^·^@PLT, as described as above. The images of nano NO^·^ micelles were obtained using a Leica DM400 B LED (Leica, Germany) fluorescence microscope. These images were used to prove the presence of NO^·^ in the NO^·^@PLT. Fluorescently labeled nano NO^·^ micelles were used to prove the loading behavior in NO^·^@PLT.

Western blotting was performed to detect the characteristic membrane proteins of PLTs. Nano NO^·^ micelles and NO^·^@PLT were separated and transferred to a PVDF membrane after 6% SDS-PAGE gel electrophoresis. The PVDF membrane was sealed with BSA-containing TBST at room temperature for 2 h. CD41-specific antibodies (1:1000) were added to the membrane and incubated at room temperature for 4 h. After the PVDF membrane was washed, IgG-specific antibodies (1:5000) were added and incubated at room temperature for 2 h. The ECL luminescent liquid was prepared by mixing liquid A and liquid B (1:1) and added to the PVDF film placed in the visualizer. After exposure to this liquid, the strip could be observed. An EPR spectrometer was also used to detect the magnetic properties of NO^·^ and NO^·^@PLT.

### In vitro drug release of NO^·^@PLT

U87 cells were seeded in 24-well plates at a density of 5 × 10^3^/cm^2^ cells per well for 24 h. The culture media were extracted and added into 1mL NO^·^@PLT solution (0.1 mg/mL calculated by NO^·^). SEM was applied to evaluate the morphological surface changes of the NO^·^@PLT. The NO^·^ concentration of each sample was also measured by X-band EPR at 2, 5, 10, 20, 30, 40, 50, and 60 min. All analyses were performed in triplicate. The percentages of nano NO^·^ micelles released from NO^·^@PLT were calculated.

### In vitro biosafety of NO^·^@PLT

When determining the hemolysis rate, nano NO^·^ micelles and NO^·^@PLT were used as the experimental group, distilled water was used as the positive control, and 0.9% saline was used as the negative control. Blood from the SD rats was collected into an anticoagulation tube, diluted to a 2% concentration, and used as the test object. Nano NO^·^ micelles and NO^·^@PLT were diluted to 1.0 mg/mL (calculated by NO^·^) with PBS. The mixture was incubated at 37 °C for 2 h and centrifuged at 1000 rpm. The absorbance of the supernatant was measured at 545 nm by using a microplate reader (Bio-Rad Laboratories Inc., Hercules, CA, USA). The hemolysis rate of each sample was calculated as follows: hemolysis rate = (Am − A0)/(A1 − A0) × 100%. Am was the absorbance value of each detected concentration on the microplate reader. A1 was the positive control, whereas A0 was the negative control.

To mimic the effect of intravenous administration of NO^·^@PLT on vascular endothelial cells, a cytotoxicity test was conducted as previously described [35]. The cell monolayers were washed with PBS, and 100 µL RPMI media (not supplemented) was added to each well and incubated at 37 °C under 5% CO_2_ for 1 h. The cells were then treated with nano NO^·^ micelles or NO^·^@PLT at 0.1, 0.2, 0.5, 1.0, and 2.0 mg/mL concentrations and incubated at 37 °C for 24 h. Monolayers in the growth media were used as negative controls. To detect cell viability, the CCK8 assay was performed following the manufacturer’s protocol.

### Construction of tumor model in nude mice

The animal care protocol was approved by the institutional animal care and use committee. Five-week-old male BALB/c nude mice were kept in the animal center of Nantong University, and the U87 cell suspension (200 µL, 1 × 10^6^ cells per mouse) was implanted into the left axilla of the mice. Tumors were allowed to grow for 2 weeks. Nontumor-bearing rats served as negative controls.

### Target behavior of NO^·^@PLT in vitro and in vivo

In order to track nano NO^·^ micelles, the FITC were added into the obtained acetal-PEG-b-PCMS-NO^·^. Afterward, the FITC labeled NO^·^@PLT was constructed as described as above. The targeting behavior of NO^·^@PLT was investigated both in vitro and in vivo. In the in vitro experiments, a modified Transwell system was used. The U87 cells were incubated in the bottom layer at 37 °C under 5% CO_2_ for 24 h. FITC-labeled NO^·^@PLT were added to the upper layer for 1 h, and the cells were scanned using a confocal laser to generate fluorescence images. A Leica DM400 B LED fluorescence microscope (Leica, Germany) was used for imaging.

To demonstrate the effectiveness of PLTs as carriers for drug delivery to tumors, they were labeled with Cy5 and used to prepare the NO^·^@PLT. Cy5 was added to the glutamic acid solution and incubated at 37 °C for approximately 4 h in the dark. Afterward, the Cy5-labeled nano NO^·^ micelles were separated from free Cy5 through 24 h of dialysis and used for in vivo imaging. The PerkinElmer IVIS Lumina Series III *ex/in vivo* imaging system (Waltham, MA, USA) was used for imaging. Three mice were sacrificed 1.5 h after NO^·^@PLT injection, and major organs, including the heart, liver, spleen, lung, and kidney, and the tumor were collected for ex vivo imaging.

### Imaging features of NO^·^@PLT in vitro

The imaging features of NO^·^@PLT were investigated in vitro through MRI. The experiments were conducted using a 3.0-T MR scanner (Signal 750 w, GE Healthcare, USA) with a 24-channel head matrix coil. T1WI and T2-weighted imaging (T2WI) sequences were used for MRI. The parameters for T1WI were as follows: the echo time (TE) was 9.924 ms, the repetition time (TR) was 568 ms, the layer spacing is 2.7 mm and the field of view (FOV) was 6 cm. In T2WI sequence, TE was 91.256 ms, TR was 5075 ms, layer spacing was 2.7 mm and FOV was 8 cm. Agar (1.5 g/mL) was used as a control. The concentration was 200 × 10 ^9^/L for PLTs, 1.0 mg/mL for nano NO^·^ micelles, and 1.0 mg/mL for NO^·^@PLT (calculated by NO^·^). To reduce the number of tribromoethanol-induced deaths, as little anesthetic as possible was administered to U87 tumor-bearing mice before each MRI scan.

To compare the simulation with the normal tissue in 3 dimensions, intuitive, stereoscopic and high-quality 3D images were performed using double-energy spiral computed tomography (CT). The imaging experiments were performed using cannulas. Agar was used to simulate a normal human body and was packed into a 15-mL tube. Agar, PLT, nano NO^·^ micelles, or NO^·^@PLT was packed into a 5-mL tube. The 5-mL tube was then placed into the 15-mL tube, which was scanned using a double-energy spiral CT scanner (Somatom Force, Siemens, Germany) to obtain a three-dimensional image, and MRI was performed for coronal images. The scanning parameters for CT were as follows: voltage, 80 kV; current, 164 mA; window width, 300; window level, 50; layer thickness, 2 mm; layer spacing, 2 mm; and voxel size, 0.488*0.488. The scanning parameters of the 3.0-T MR scanner were the same as those used before.

To explore the relationship between NO^·^@PLT concentrations and MRI, different NO^·^@PLT concentrations were added to a 6-well plate, and the parameters of MRI are the same as previous experiments.

### MRI experiments of NO^·^@PLT in vivo

MRI experiments of NO^·^@PLT were conducted in vivo by using tumor-bearing rats. The rats were randomized into four groups (PBS, PLTs, nano NO^·^ micelles, or NO^·^@PLT), and 150 µL of PLTs, nano NO^·^ micelles, or NO^·^@PLT (1.0 mg/mL) was injected via the tail vein into the rats in the three groups. After injections, the rats in these three groups were euthanized at different time points (5 min, 1.5 h, 2.5 h, and 3.5 h). The remaining group of tumor-bearing rats was injected with only saline solution and used as controls. The 3.0-T MR scanner was also used for all MR scans in vivo. The parameters for the T1WI were as follows: TE of 10.292 ms, TR of 518 ms, layer spacing of 2.7 mm, and FOV of 6 cm. The liver and kidneys of each nude mouse were also observed for changes.

### Histological examination

Histological examination was conducted after NO^·^@PLT (150 µL, 1.0 mg/mL calculated by NO^·^) administration. The heart, liver, spleen, lungs, kidneys, and brain were collected at 7, 14, and 30 days, fixed with paraformaldehyde for 48 h at 4 °C, embedded in paraffin, and stained with hematoxylin and eosin. The slices were observed using an optical microscope.

### Hematology and biochemical experiments

Hematology and biochemical experiments were conducted after NO^·^@PLT administration for 7 days. Blood biochemistry analysis was performed using blood samples and an automated biochemical analyzer. Biochemical indices of liver functions (ALT and AST) and kidney functions (BUN and CREA) were evaluated using enzyme-linked immunosorbent assay (ELISA) quantification kits. Inflammatory index markers (IL-6 and TNF-α) were measured through ELISA.

### Statistical analysis

Statistical analysis was performed using SPSS software version 20.0. The data are expressed as the mean ± SEM. T tests were used for comparisons between two groups, and ANOVA was used for multigroup comparisons. *P* < 0.05 was considered significant.

## Results and discussion

### **Synthesis and characterization of NO**^·^**@PLT**

**Fig. 1:**
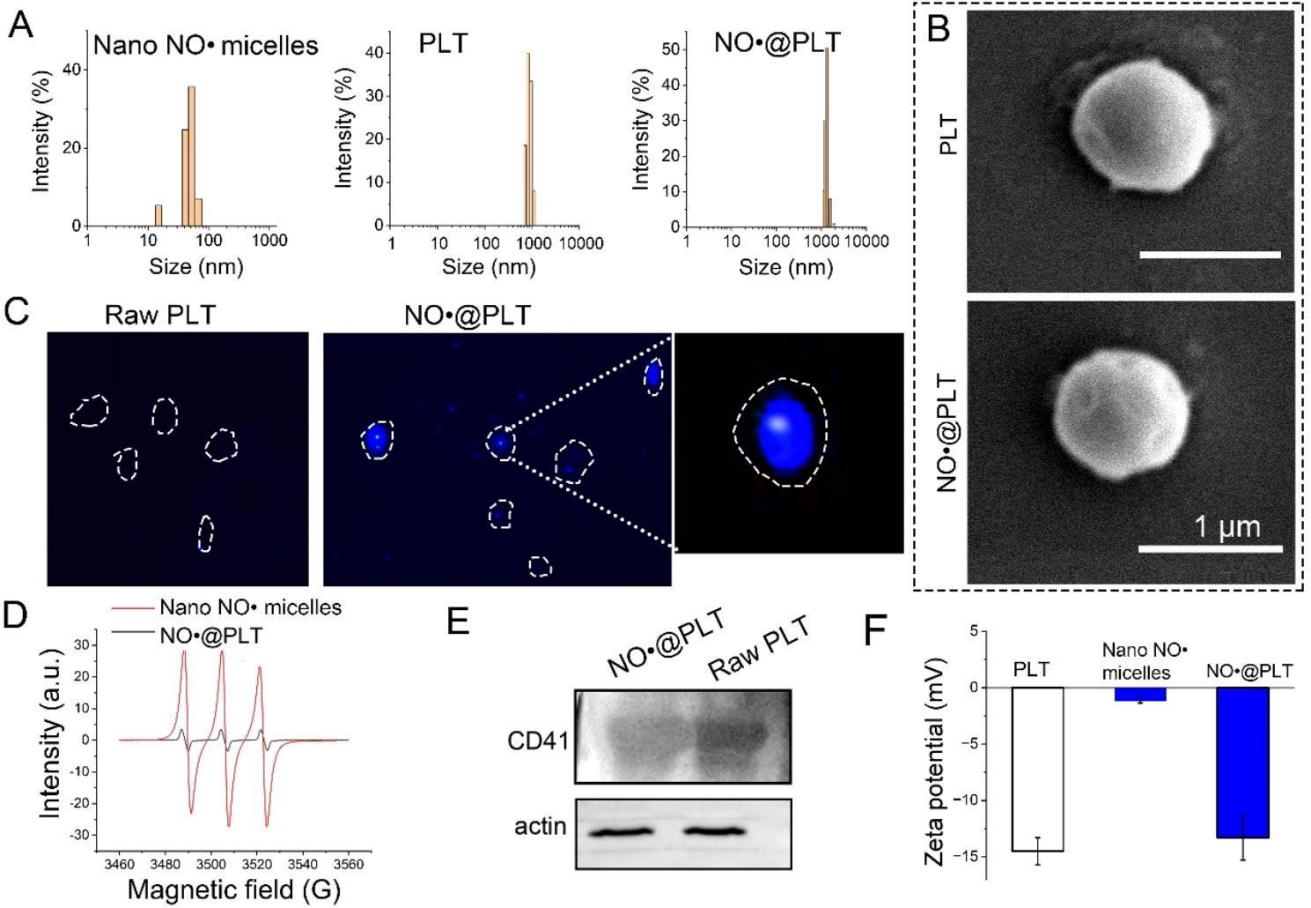
Synthesis and characterization of the NO^·^@PLT. (**A**) Size distribution of nano NO^·^ micelles, PLT, and NO^·^@PLT. (**B**) SEM images of PLTs and NO^·^@PLT. Scale bar = 1 μm. (**C**) Fluorescence staining images of NO^·^@PLT and PLTs (without nano NO^·^ micelles). (**D**) Magnetic detection images of nano NO^·^ micelles and NO^·^@PLT. (**E**) Western blotting experiment of PLTs and NO^·^@PLT. (**F**) Zeta potential changes during the synthesis of NO^·^@PLT

PLTs are natural drug carriers because of their ability to circulate in the bloodstream and accumulate at tumor sites. Researchers have recently developed various strategies to load drugs onto PLTs, including physical adsorption, chemical conjugation, and encapsulation [36–38]. Our previous study proved that ultrasound can improve the permeability of PLT membranes because of sonoporation [31–33]. Ultrasound-formed tiny pores on the PLT membranes could enable the passive entry of drug molecules into cells [39]. At first, the core-shell-type nano NO^·^ micelles from the obtained block copolymers were obtained (schematic of the structure was shown in Fig. S1) and the average particle size in PBS (pH 7.4) was 51.3 ± 3.8 nm (Fig. 1A), which is favorable for PLT loading. In the process, ultrasound (80 W, 40 kHz) was used to aid the entry of nano NO^·^ micelles into PLTs. Furthermore, the PGE1 was added to act as an aggregation inhibitor of PLT, reduce hyperreactivity and inhibit activation. Because of the use of mild sonication and aggregation inhibitors in the loading process, the original morphological features of the PLT carriers were maintained, as the diameter was similar to that of raw PLTs (range: 687–1035 nm). Furthermore, the outer surface exhibited a slightly rough morphology compared with that of raw PLTs (Fig. 1B). Next, we characterized the NO^·^@PLT with a fluorescent probe (Fig. 1C). Each NO^·^@PLT (outline was marked with a dashed white line) had blue fluorescence, which indicated the presence of nano NO^·^ micelles. Because NO^·^ has magnetic properties, the EPR experiments were performed. As shown in Fig. 1D, NO^·^ exhibited a triplet curve in the EPR spectrum, and the magnetic properties of NO^·^@PLT were examined using the same procedure. The spectral image of NO^·^@PLT also exhibited a triple curve similar to that exhibited by the spectral image of NO^·^.

Furthermore, western blotting was performed to detect the CD41 feature protein (one of the characteristic proteins of PLTs) in NO^·^@PLT. The results (Fig. 1E) proved the existence of PLTs in the NO^·^@PLT. During the NO^·^@PLT preparation process, the NO^·^ micelles were encapsulated in the PLTs as supported by the measurement of the membrane zeta-potentials (Fig. 1F). The aforementioned data displayed that the NO^·^@PLT had been successfully fabricated, with PLTs as delivery vehicles for NO^·^.

PLTs not only are crucial players in hemostasis and thrombosis but also have the potential to serve as drug delivery vehicles for targeted cancer therapy [40–42]. The use of PLTs as drug delivery vehicles principally benefits from their unique ability to recognize and interact with tumor cells. The expression of specific surface receptors on both PLTs and tumor cells facilitates this recognition. One such receptor is P-selectin, which interacts with PSGL-1 on tumor cells, thereby promoting PLT–tumor cell interactions [43]. Additionally, PLTs express glycoprotein Ib-IX, which binds to the von Willebrand factor exposed on the surface of activated endothelial cells and tumor cells [44]. In this study, western blotting was performed to detect the PLT-specific protein CD41. The NO^·^@PLT were positive for CD41. This indicates that PLTs that serve as NO^·^ carriers are CD41-expressing PLTs and have the ability to target delivery of their content. Then, the targeted behavior of NO^·^@PLT was studied.

### **Targeted behavior of NO**^·^**@PLT**

**Fig. 2:**
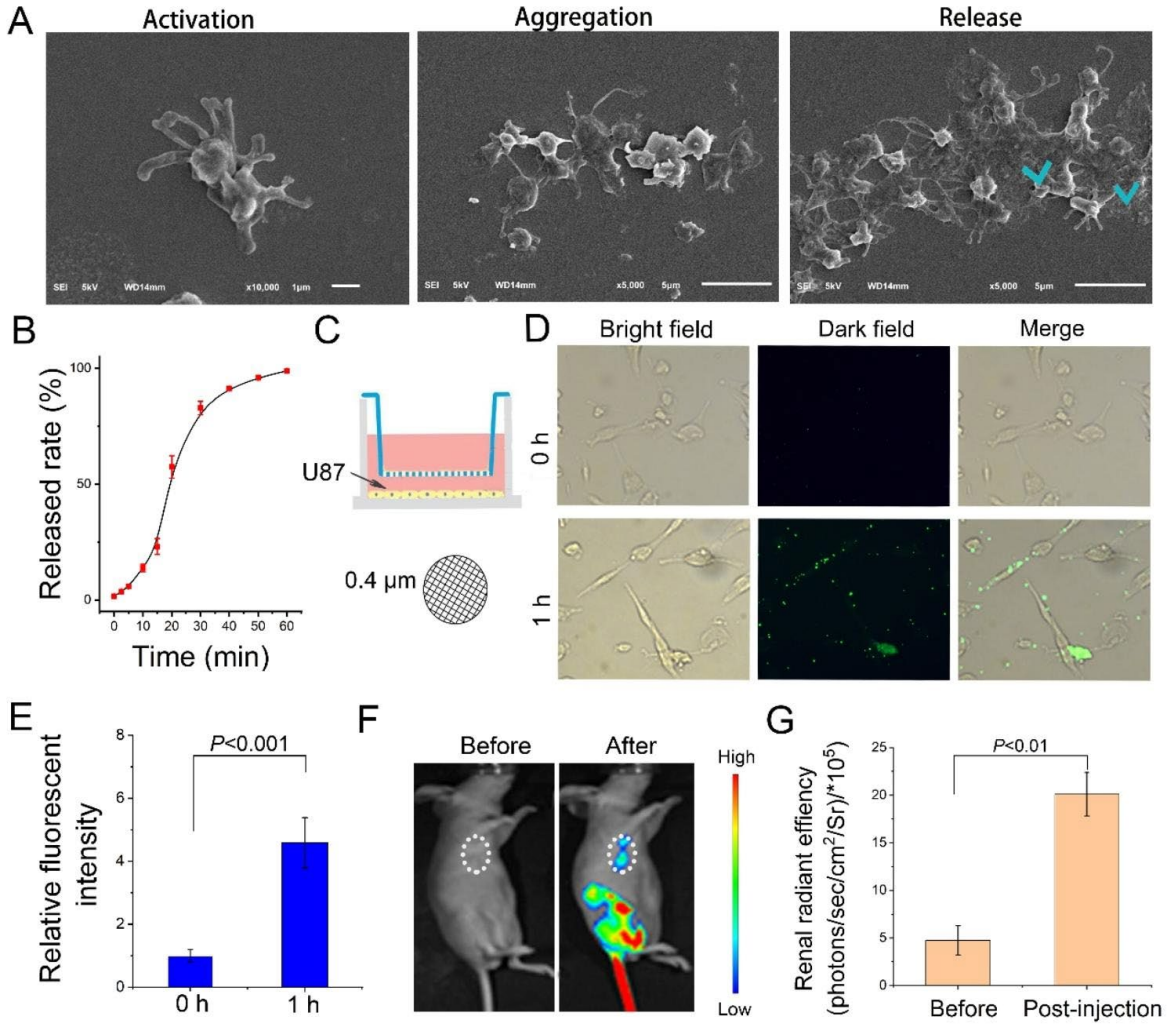
Target behavior of NO^·^@PLT in vitro and in vivo. (**A**) SEM images of NO^·^@PLT during the activation process. (**B**) The nano NO^·^ micelles release curve from NO^·^@PLT during the activation. (**C**) Experimental schematic of the Transwell system (0.4-µm pore size). The NO^·^@PLT was added to the upper layer, and U87 cells were cultured in the lower layer. (**D**) Fluorescence images of U87 cells in the lower chamber. NO^·^@PLT was modified with Cy 5.5. (**E**) Fluorescence analysis of fluorescently labeled NO^·^@PLT in the lower layer. (**F**) In vivo images of mice bearing U87 tumors treated with NO^·^@PLT at 0 h (before) and 1.5 h (after). (**G**) Tumor site in vivo fluorescence intensity quantification after intravenous injection at 0 h (before) and 1.5 h (after). Data are presented as the mean ± SD (n = 5)

Target delivery of nano NO^·^ micelles to the tumor site and its enrichment, which are crucial, were investigated here. Then, we tested the targeted behavior of NO^·^@PLT. Once PLTs recognize tumor cells, they undergo activation and aggregation. This involves the release of their contents from PLT granules. NO^·^@PLT were activated by tumor cells, as its deformation appeared (3–8 tentacles appear). Then the activated PLTs aggregated other NO^·^@PLT (Fig. 2A) through tentacles contact. Finally, nano NO^·^ micelles in the NO^·^@PLT were released. Together, this deformation enhanced the activating effects of NO^·^@PLT. Approximately 90% of nano NO^·^ micelles were released over 40 min after the NO^·^@PLT were cocultured with the U87 cells (Fig. 2B).

To further demonstrate the good targeting effect of PLTs on tumor cells, an improved Transwell experiment was conducted. The fluorescently labeled NO^·^@PLT were added to the upper layer, and the Transwell system could only allow particles of < 400 nm to pass through (Fig. 2C). With time, the NO^·^@PLTs were activated and release their contents. The mean particle size of released nano NO^·^ micelles was about 217.4 nm, which was small enough to pass through the Transwell system (Fig. S2). Interestingly, the PLT drug carriers also helped nano NO^·^ micelles identify tumor cells and adhere to the cell periphery region (Fig. 2D). The fluorescence intensity increased significantly (*P* < 0.001, Fig. 2E).

The excellent in vitro results prompted us to hypothesize that NO^·^@PLT has a good potential to deliver NO^·^ in vivo. The signal intensity of MRI was markedly affected by the selective enrichment of NO^·^ within the tumors. Therefore, the NO^·^@PLT biodistribution was investigated through intravenous injection of FITC modified NO^·^@PLT into BALB/c nude mice bearing U87 xenograft tumors. At 1.5 h after injection of NO^·^@PLT, the fluorescence signals from the tumors increased significantly compared with those before the injection (Fig. 2F). The relative fluorescence intensity increased significantly (*P* < 0.01, Fig. 2G).

### Evaluation of treatment safety

**Fig. 3:**
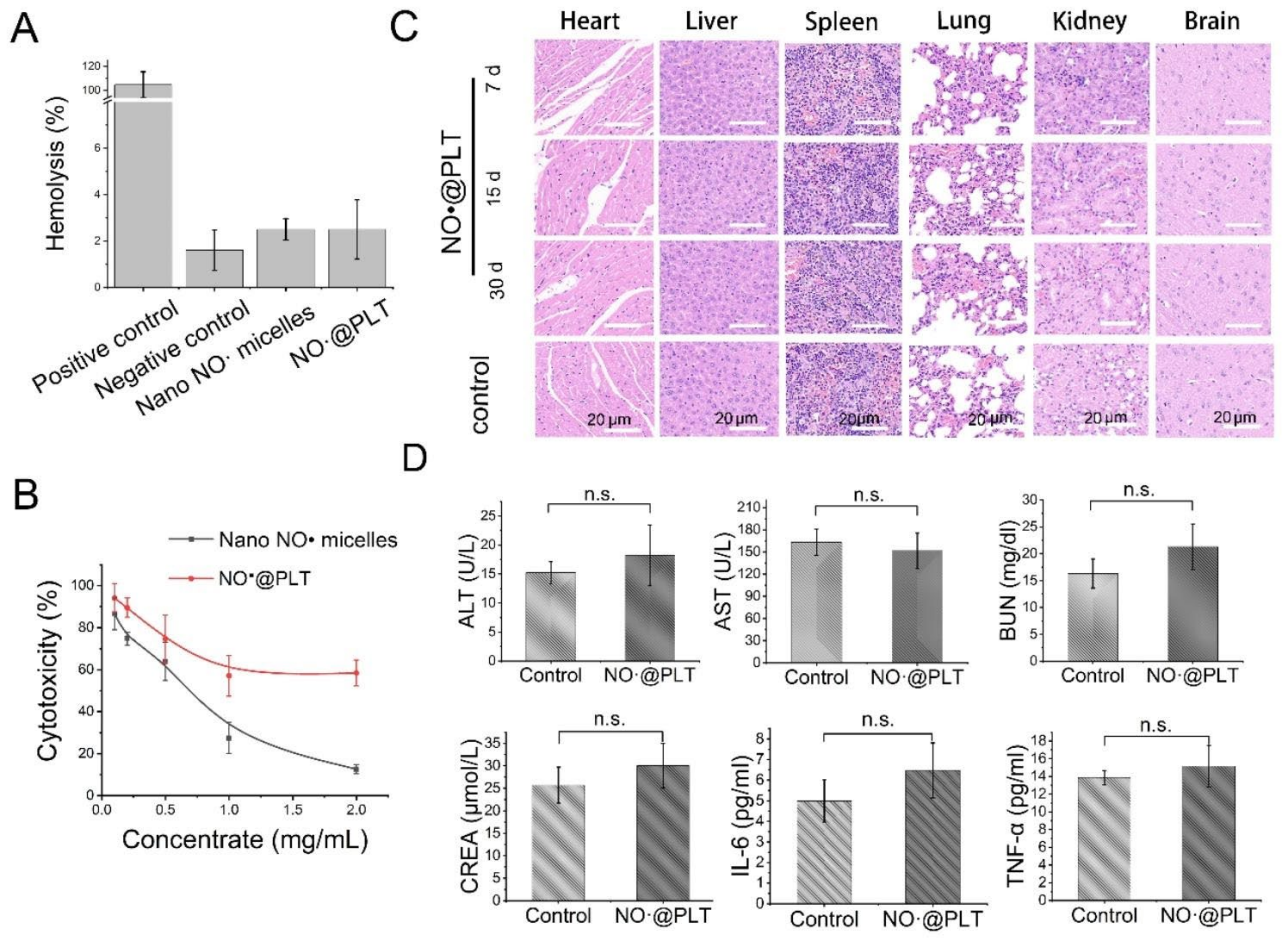
Biosafety of NO^·^@PLT. (**A**) The hemolysis rate of nano NO^·^ micelles and NO^·^@PLT. Distilled water was used as the positive control, and 0.9% saline was used as the negative control. (**B**) Cell cytotoxicity of nano NO^·^ micelles and NO^·^@PLT. (**C**) HE staining images of the tissue sections after injection of NO^·^@PLT from the tail vein on mice. The mice treated with PBS were used as controls. Scale bar = 20 μm. (**D**) Measurement of biochemical blood indexes, including the levels of liver function (ALT and AST), kidney function (BUN and Cr), and inflammatory index (IL-6 and TNF-α) markers on day 28 in the NO^·^@PLT treatment group. No significant difference, n.s

Drug safety is of utmost importance in drug construction and delivery [45]. ensuring that drugs are safe and effective is essential before they are made available to patients. Therefore, a safety evaluation of NO^·^@PLT was performed. As NO^·^@PLT was administered through intravenous injection, a hemolytic test of NO^·^@PLT was performed. The hemolysis rate of NO^·^@PLT did not exceed 5% (Fig. 3A). Based on the hemolytic rate, biomaterials with the hemolysis rate less than 5% can be considered as feasible blood-contacting materials [46]. The results showed that NO^·^@PLT may be used safely for intravenous administration.

After intravenous injection of NO^·^@PLT, they circulated in the blood vessels for a long time. Therefore, the stimulatory effect of NO^·^@PLT on vascular endothelial cells also needs to be evaluated. The viability of vascular endothelial cells was inhibited and their cytotoxicity increased as the concentration of NO^·^ particles or NO^·^@PLT increased (Fig. 3B). As delivery vehicles, PLTs in NO^·^@PLT could alleviate the stimulatory effect of nano NO^·^ micelles on vascular endothelial cells. Based on safety considerations, NO^·^@PLT with 1.0 mg/mL nano NO^·^ micelles were used in the following studies.

The long-term toxicity of NO^·^@PLT (1.0 mg/mL, determined by NO^·^) was next assessed in normal mice. 500 µL of blood samples were taken at various time intervals after the mice had received intravenously given doses of NO^·^@PLT through the tail vein in order to examine the drug’s effects on organs and tissues. Several important organs, including the heart, liver, spleen, lungs, kidney, and brain (Fig. 3C), showed no evident interstitial fibrosis or inflammatory cell aggregation. The blood biochemistry in the mice during the treatment period, comprising the levels of liver function enzymes (ALT and AST), kidney function parameters (BUN, Cr), and inflammatory index markers (IL-6 and TNF-α), did not alter substantially from that in the control group (Fig. 3D). The innovative “metal-free” MRI contrast agents (NO^·^@PLT) were suitably safe for the human body, according to these datas.

Many studies have been done to modify existing metal MRI contrast agents in order to improve their biosafety. Lu et al. proposed a new concept of organogadolinium macrochelates constructed from the coordination between Gd^3+^ and macromolecules. It showed that Gd^3+^ would not release indirectly, ensuring biosafety for in vivo applications [6]. The other metal-containing contrast agents (such as Cu and Mn) also could be modified for improving their stability, safety, and biocompatibility [11, 47–50]. As we know, several factors contribute to the biotoxicity of MRI contrast agents. A major concern is the heavy metals, such as gadolinium, present in some contrast agents. These metals can accumulate in the body and may cause toxicity over time [51]. In this study, NO^·^@PLT is a metal-free contrast agent that eliminates the risk of toxic effects associated with traditional metal-based agents. Thus, these make MRI contrast agent (NO^·^@PLT) is safer for patients, especially those with an impaired renal function.

### In vitro MRI of NO^·^@PLT

**Fig. 4:**
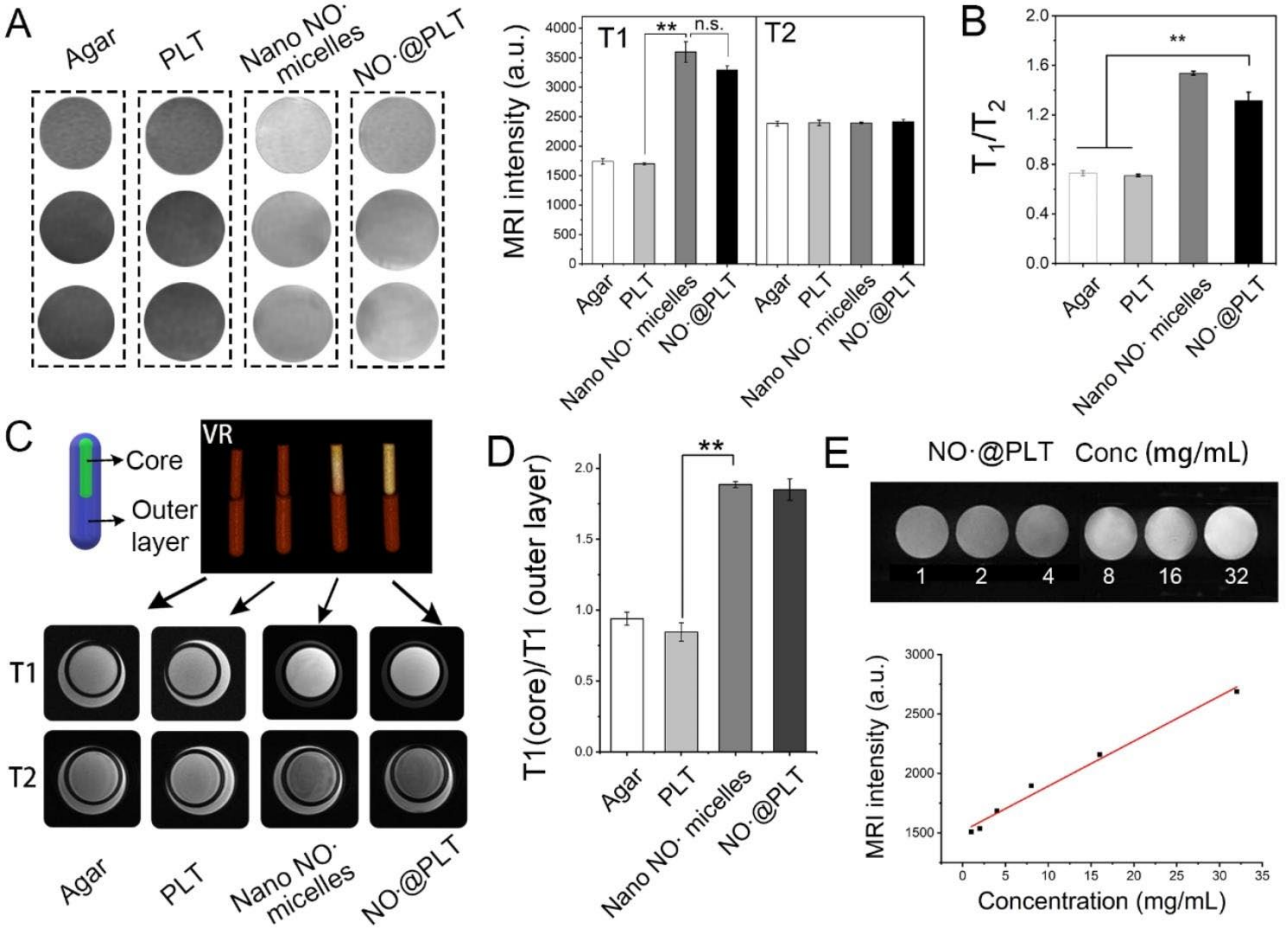
In vitro MRI of NO^·^@PLT. (**A**) T1-weighted images (T1WI). The concentration was 0.015 g/mL for Agar, for PLT, 1.0 mg/mL for nano NO^·^ micelles, and 1.0 mg/mL for NO^·^@PLT (calculated by nano NO^·^ micelles). (**B**) The changes in signal intensity as indicated by T1WI/T2WI of panel (**A**). (**C**) 3D image of tube and MR images of agar, PLT, nano NO^·^ micelles, and NO^·^@agar. (**D**) The ratio of signal intensity in T1WI (core)/ (outer layer) in each group. (**E**) Correlation of NO^·^@PLT concentration with T1WI signal intensity (the inset shows the signal intensity in T1WI of NO^·^@PLT with different concentrations). **P < 0.01. No significant difference, n.s

T1WI and T2WI are the two most commonly used sequences of MRI in clinical practice. The imaging performance of NO^·^@PLT was measured using an MRI scanner. NO^·^@PLT exhibited contrast enhancement in T1WI (Fig. 4A), which was similar to that of NO^·^ micelles. Moreover, no significant difference was observed in T2WI (Fig. S3). Then, the ratio (T1WI/T2WI) of NO^·^@PLT was calculated to be ≈ 1.32 (Fig. 4B), indicating that NO^·^@PLT can be used as a T1WI mode MRI contrast agent.

As an MRI contrast agent, NO^·^@PLT was expected to enhance the signal, which aids in differentiating the diseased tissues from the surrounding normal tissue. To validate the predictive performance of NO^·^@PLT, a phantom made of agar was used to mimic a normal human body. As shown in Fig. 4C, the agar (negative control), PLTs, nano NO^·^ micelles (positive control), or NO^·^@PLT was added to a small tube and then inserted into a large tube. The T1WI signal intensity in the core of the NO^·^ micelles and NO^·^@PLT groups were markedly higher than those in the core of agar and the PLT group (*P* < 0.05, Fig. 4D). Furthermore, the nano NO^·^ micelles, loaded in the PLT, did not seem to affect their magnetic resonance imaging, as there was no difference in T1WI between nano NO^·^ micelles and NO^·^@PLT. Unsurprisingly, little difference in T2WI was observed between the NO^·^@PLT and other groups (Fig. S4). Thus, the T1WI/T2WI ratios were high in the NO^·^ and NO^·^@PLT groups (Fig. S5). Therefore, the MR images acquired after injection of NO^·^@PLT were believed to exhibit significant signal differences between gliomas and other peripheral normal tissues.

In addition, the NO^·^ concentration in the glioma is also a key factor, as we noticed that the T1WI signal intensity versus the NO^·^@PLT concentration exhibited a linear relationship (Fig. 4E). Hence, improving the NO^·^@PLT concentration in the glioma is an effective method for obtaining good image quality in MRI.

### Biodistribution of NO^·^@PLT in vivo

Based on the excellent in vitro results, we hypothesized that PLTs have good potential to deliver nano NO^·^ micelles in vivo to treat tumors. The selective concentration of nano NO^·^ micelles within tumors has a significant impact on the quality of the MRI image. Therefore, we studied NO^·^@PLT biodistribution through intravenous injection of NO^·^@PLT into BALB/c nude mice bearing U87 xenograft tumors.

**Fig. 5:**
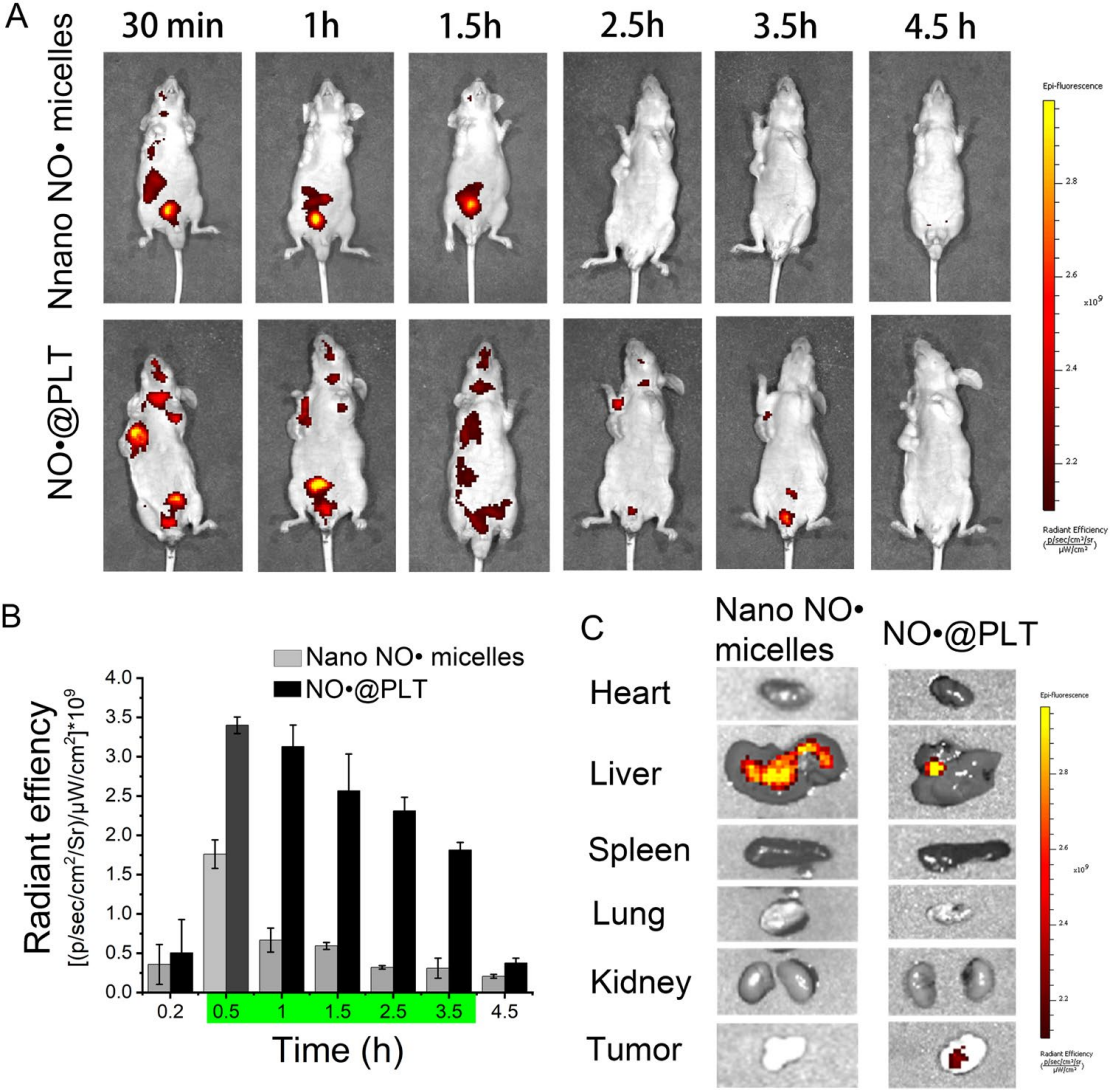
In vivo imaging assessment of the biodistribution of NO^·^@PLT. (**A**) In vivo images of mice bearing U87 tumors treated with NO^·^@PLT and free nano NO^·^ micelles particles after intravenous injection. (**B**) Fluorescence intensity quantification of tumors after treatment with NO^·^@PLT or free nano NO^·^ micelles particles. (**C**) In vivo image of Cy5 accumulation in the tumor and other organs in the nano NO^·^ micelles and NO^·^@PLT groups

Fluorescence signals from the tumors in the NO^·^@PLT group greatly outperformed those from the free nano NO^·^ micelles group at 0.5 h after injection (Fig. 5A). At 3.5 h after injection, the NO^·^@PLT group continued to display tumor fluorescence, whereas no fluorescence accumulation was observed from the tumor site in the free nano NO^·^ micelles group. The fluorescence intensity in tumor tissues from the NO^·^@PLT group was significantly higher than that from the free nano NO^·^ micelles group from 0.5 to 3.5 h (Fig. 5B). At 3.5 h, in both groups, fluorescence signals were barely detectable in the lungs, spleen, and heart. Significantly more NO^·^@PLT accumulated in the tumor than free nano NO^·^ micelles, while the liver and kidney exhibited nano NO^·^ micelles clearance (Figs. 5C and S6–S11). Therefore, we could conclude that the most effective treatment duration for NO^·^@PLT was 30 min, and NO^·^@PLT lost efficacy after 3.5 h.

According to the aforementioned results, PLTs could serve as nano NO^·^ micelles carriers for targeted cancer drug delivery. This may be due to their unique ability to recognize tumor cells, release bioactive molecules, form a protective shield, and deliver therapeutic agents. As observed previously, PLTs can be loaded with therapeutic agents and used as drug delivery vehicles for targeted cancer therapy [52, 53]. By modifying PLT surfaces with specific ligands or antibodies, drug-loaded PLTs can be made to selectively bind to tumor cells, thereby delivering the therapeutic payload directly to the tumor site [54]. Furthermore, PLT drug carriers can overcome the poor in vivo stability of nitroxide-based MRI contrast agents, as the NO^·^@PLT showed good anti-interference performance (Fig. S12). The PLT-based targeted drug delivery approach minimizes off-target effects and improves the efficacy of anticancer drugs.

### **Imaging of glioma by using NO**^·^**@PLT as contrast agent**

Because NO^·^@PLT exhibited excellent tumor-targeting ability and could more effectively differentiate between tumor and normal tissues, we sought to further evaluate NO^·^@PLT in the U87 subcutaneous model. We thus evaluated the capabilities of NO^·^@PLT to serve as MRI contrast agent and help detect brain glioma.

**Fig. 6:**
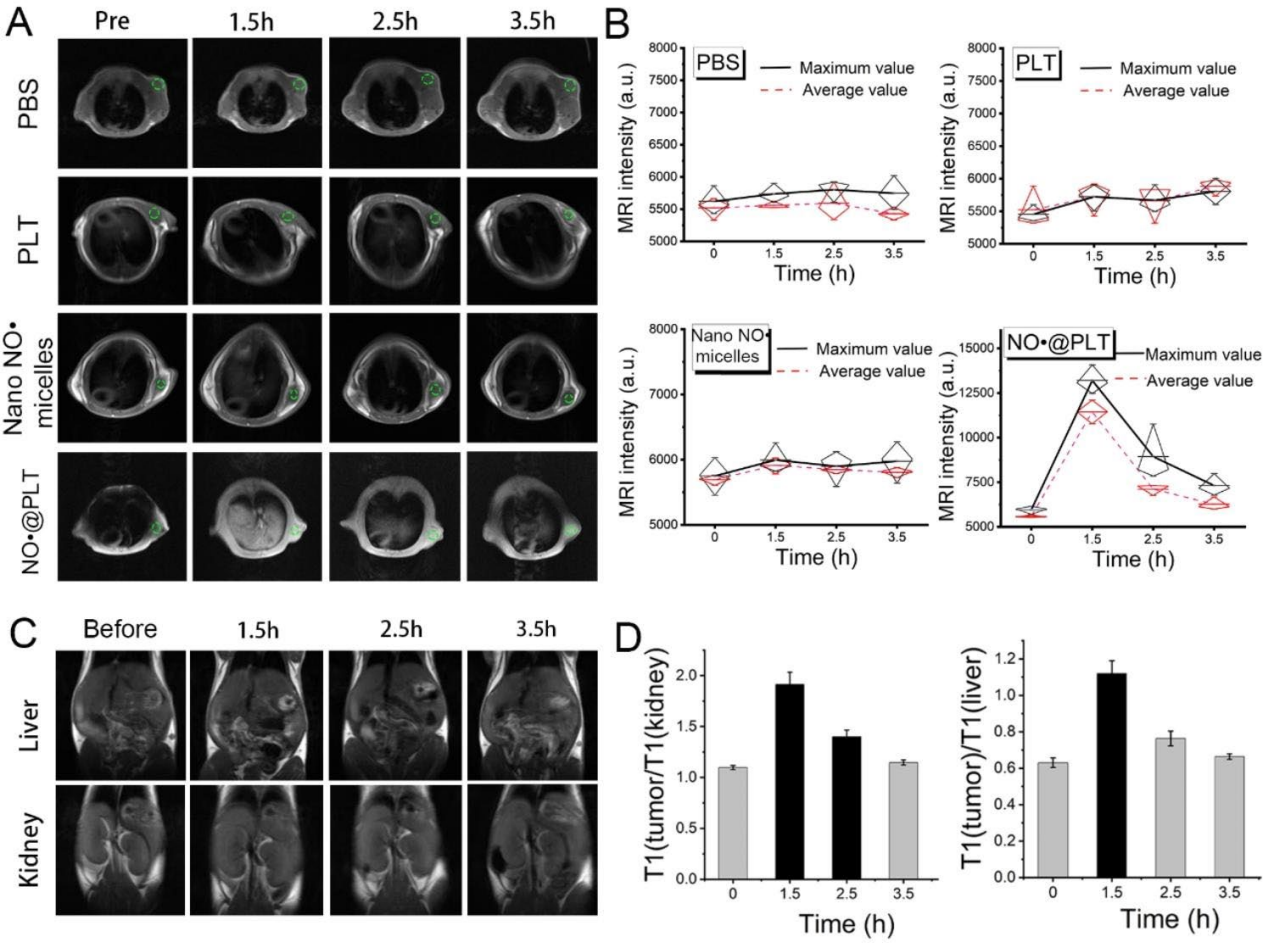
Application of NO^·^@PLT in the subcutaneous glioma (U87) diagnosis. (**A**) The images of subcutaneous glioma in T1WI by using PBS (control), PLTs, nano NO^·^ micelles, and NO^·^@PLT as contrast agents. (**B**) Quantitative analysis of the T1WI signal intensity of a subcutaneous glioma. (**C**) Images of the liver and kidney in T1WI after NO^·^@PLT administration. (**D**) Quantitative analysis of the T1WI signal intensity of the liver and kidney after NO^·^@PLT administration

NO^·^@PLT was intravenously administered to the mice. They could readily enhance the contrast in the tumor region in T1WI (Fig. 6A). The signal intensity (mean or maximum) in T1WI in the PBS, PLT, or NO^·^ groups exhibited no significant changes (Fig. 6B). Meanwhile, the intensity in T1WI from NO^·^@PLT was significantly stronger in the tumor site, and the intensity was also significantly enhanced by 1.74 times (maximum intensity) at 1.5 h after drug administration, compared with the intensity before administration. The signal intensity in the NO^·^@PLT group was also significantly higher than that in the control group (PBS) (Fig. S13).

The tumor pattern delineated by the NO^·^@PLT-enhanced MRI was clear from 5 min to 3.5 h, whereas signal intensity in tumor returned to normal by 4.5 h after injection (*P* < 0.001, Figs. S14 and S15).

Although in vitro studies have shown that nano NO^·^ micelles can be used as an MRI contrast agent (Fig. 4), no significant change in MRI signal intensity was observed in the nano NO^·^ micelles administration group in the in vivo studies. This may be due to low local nano NO^·^ micelles concentrations in the tumor because of the systemic distribution of nano NO^·^ micelles or its clearance by the blood. With the help of the PLT drug delivery system, nano NO^·^ micelles could accumulate at the tumor site, and it demonstrated good MRI contrast enhancement. These results indicated that NO^·^@PLT was valuable for MRI-based glioma diagnosis.

Furthermore, the liver and kidney signal intensity did not change significantly compared with the glioma signal of the nude mouse under the same conditions (Fig. 6C and S16). This may be because the NO^·^@PLT imaging system exerts a good targeting effect on tumors, and NO^·^@PLT do not accumulate in the liver and kidney. Therefore, the signal intensity in MRI of the liver and kidneys can serve as background intensity in MRI-based tumor diagnosis. Then, compared with the intensity in T1WI in the kidney and liver, those from NO^·^@PLT in the tumor site were significantly stronger, approximately 1.95 times (kidney) and 1.08 times (liver) at 1.5 h (Fig. 6D). The aforementioned data suggested that NO^·^@PLT had excellent performance as MRI contrast agent, with an optimum time of approximately 1.5 h after administration. Brain gliomas can be diagnosed using the signal intensity in MRI before and after administration or by establishing the signal in the kidney as the background.

The results revealed that the NO^·^@PLT could overcome the low contrast and poor in vivo stability associated with nitroxide-based MRI contrast agent. However, NO^·^@PLT still exhibit the inherent disadvantages of metal-free contrast agents compared with metallic contrast agents, such as lower sensitivity and shorter half-life [55, 56]. This study revealed that the metal-free MRI contrast agent (NO^·^@PLT) is a safer and more versatile alternative to traditional metal-based agents, with comparable or superior imaging capabilities. The biocompatibility, improved image quality, and cost-effectiveness of NO^·^@PLT make it promising candidates for future clinical applications.

## Conclusion

In this study, we developed a new metal-free MRI contrast agent (NO^·^@PLT) by camouflaging NO^·^ with PLTs. Because PLTs and NO^·^ are widely present in the body, NO^·^@PLT induced less nephrotoxicity or hepatotoxicity, which indicates that these particles could serve as an alternative MRI contrast agent. As PLTs could bind to tumor cells through the interaction of PLT surface receptors with tumor cell surface markers, PLT-mediated drug delivery could lead to selective accumulation of nano NO^·^ micelles at the tumor sites. Then, the PLTs release their cargo of MRI contrast agents, which significantly improves the MRI intensity. With the high MRI intensity, the NO^·^@PLT highlighted subcutaneous brain tumors and clearly differentiated the margin between the tumor and normal tissue. The effective imaging window of NO^·^@PLT can last for at least 3 h, thereby providing sufficient time for MRI performance. This metal-free contrast agent is highly safe, exhibits excellent selectivity, and has the potential to be an alternative option for the diagnosis of brain glioma.

### Electronic supplementary material

Below is the link to the electronic supplementary material.

**Additional file 1: Figure S1.** Schematic illustration of the nano NO^·^ micelles. **Figure S2.** Size distribution of nano NO^·^ micelles released from NO^·^@PLT. **Figure S3.** T2-weighted images. **Figure S4.** The ratio of T2 (core)/ T2 (outer layer) in each group. **Figure S5.** The changes of MRI value as indicated by T1/T2 of Figure 4C. **Figure S6–S11.** Fluorescence intensity quantification of heart, liver, spleen, lung, kidney and tumor after treated with nano NO^·^ micelles or NO^·^@PLT. **Figure S12.** Anti-interference ability test. **Figure S13.** The changes of MRI intensity as indicated by relative MRI intensity/ MRI intensity in PBS treated group of Figure 6A. **Figure S14.** T1-weighted MR images of subcutaneous brain tumor after NO^·^@PLT administration for 5min and 4.5 h. **Figure S15.** The changes of MRI value after NO^·^@PLT administration with time. **Figure S16.** The changes of MRI intensity in liver and kidney after NO^·^@PLT administration of Figure 6C
