# Supplementary material for: Platelets as delivery vehicles for targeted enrichment of NO· to cerebral glioma for magnetic resonance imaging
Source: J Nanobiotechnology. 2023 Dec 21;21:499. doi: 10.1186/s12951-023-02245-y (PMC10734142; doi:10.1186/s12951-023-02245-y)
Supplement: Supplementary file 1 — Additional file 1: Figure S1. Schematic illustration of the nano NO· micelles. Figure S2. Size distribution of nano NO· micelles released from NO·@PLT. Figure S3. T2-weighted images. Figure S4. The ratio of T2 (core)/ T2 (outer layer) in each group. Figure S5. The changes of MRI value as indicated by T1/T2 of Figure 4C. Figure S6–S11. Fluorescence intensity quantification of heart, liver, spleen, lung, kidney and tumor after treated with nano NO· micelles or NO·@PLT. Figure S12. Anti-interference ability test. Figure S13. The changes of MRI intensity as indicated by relative MRI intensity/ MRI intensity in PBS treated group of Figure 6A. Figure S14. T1-weighted MR images of subcutaneous brain tumor after NO·@PLT administration for 5min and 4.5 h. Figure S15. The changes of MRI value after NO·@PLT administration with time. Figure S16. The changes of MRI intensity in liver and kidney after NO·@PLT administration of Figure 6C [file 12951_2023_2245_MOESM1_ESM.docx]

Supplementary Materials for

**Platelets as Delivery Vehicles for Targeted Enrichment of NO• to Cerebral Glioma for Magnetic Resonance Imaging**

Yuchen Ding ^1^†, Min Ge ^1^†, Chao Zhang ^4^†, Juncheng Yu ^1^, Donglin Xia ^2*^, Jian He ^3*^, Zhongzheng Jia ^1*^

^1^ Department of Medical Imaging, Affiliated Hospital of Nantong University, Medical School of Nantong University, Nantong, 226001, PR China

^2^ School of Public Health of Nantong University, Nantong, 226019, PR China

^3^ Department of Nuclear Medicine, Nanjing Drum Tower Hospital, Affiliated Hospital of Medical School, Nanjing University, Nanjing, Jiangsu 210008, PR China

^4^ Department of Neurosurgery Center, Zhujiang Hospital, Southern Medical University, Guangzhou 510282, PR China

^*^ Corresponding authors. E-mails: xiadonglin@ntu.edu.cn (D.L Xia), hjxueren@126.com (J. He), [jzz2397@163.com](mailto:jzz2397@163.com) (Z.Z Jia)

† These authors contributed equally to this work.


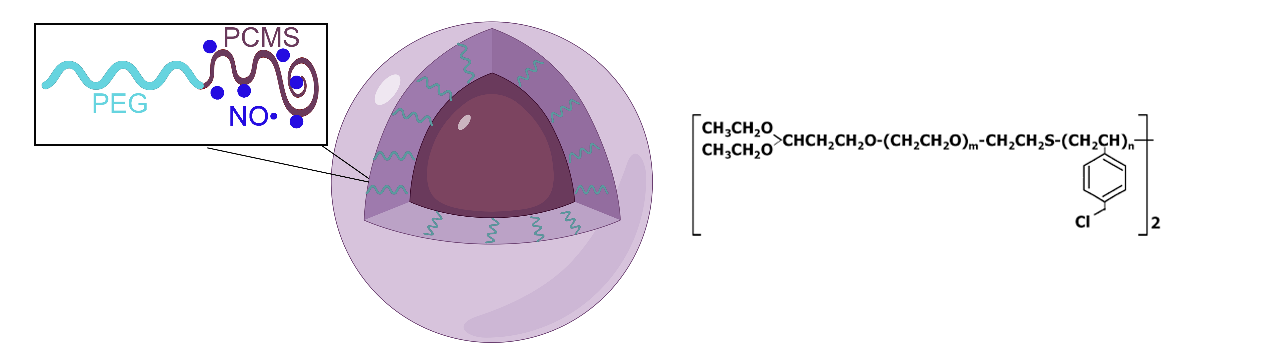


Figure S1. Schematic illustration of the nano NO• micelles.


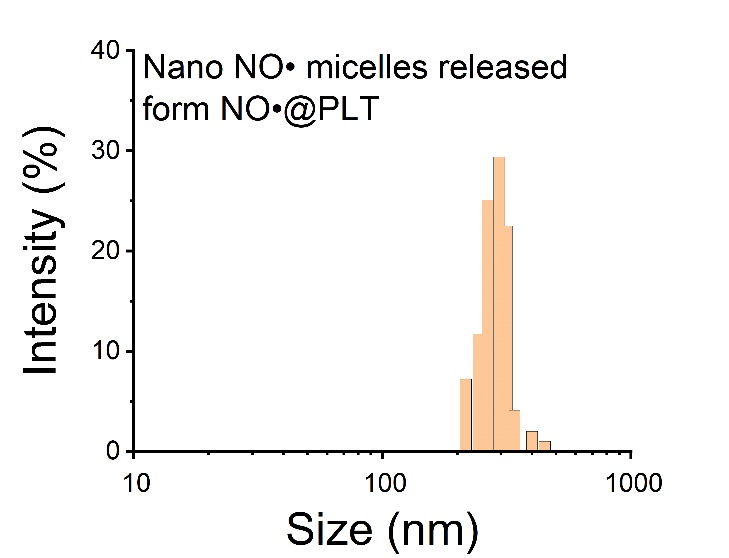


Figure S2. Size distribution of nano NO• micelles released from NO•@PLT. The average size of released NO• micelles was 217.4 ± 59.3 nm.


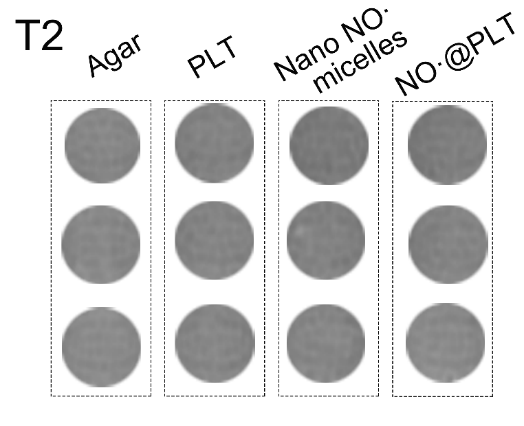


Figure S3. T2-weighted images. The concentration was 0.015 g/mL for Agar, for PLT, 1.0 mg/mL for nano NO• micelles, and 1.0 mg/mL for NO•@PLT (calculated by nano NO• micelles).


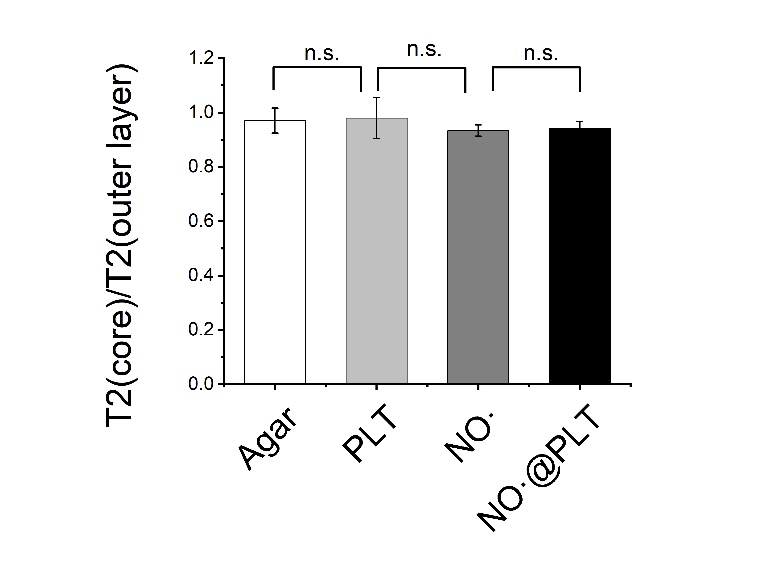


Figure S4. The ratio of T2 (core)/ T2 (outer layer) in each group. There were no much difference in T2WI between NO•@PLT and the other groups. No difference, n.s.


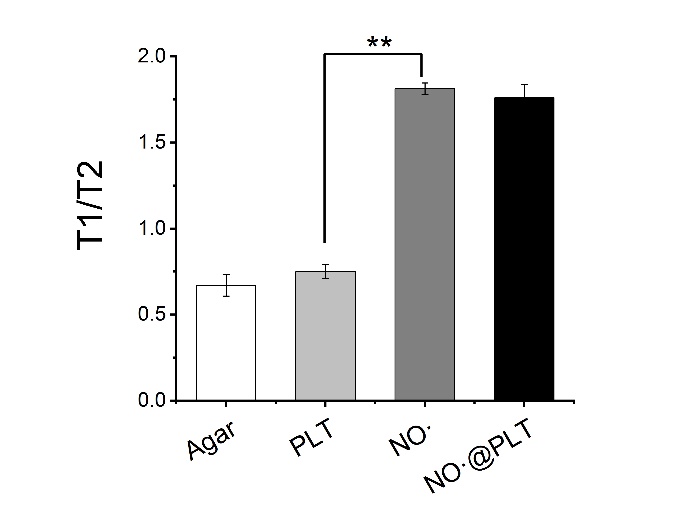


Figure S5. The changes of MRI value as indicated by T1/T2 of Figure 4C.


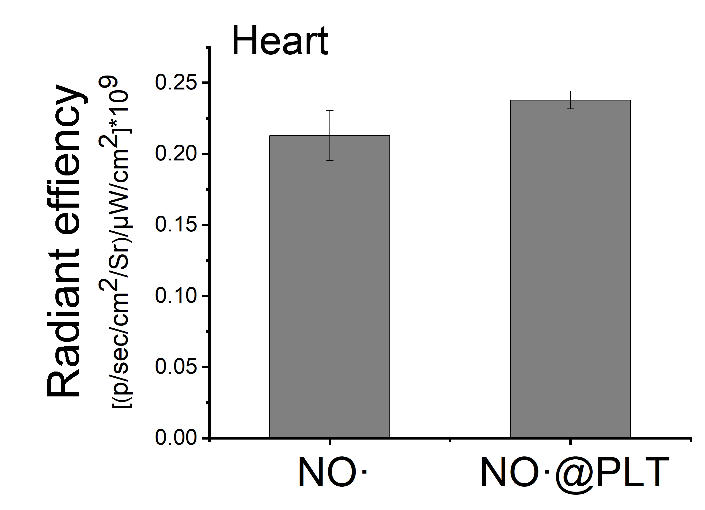


Figure S6. Fluorescence intensity quantification of heart after treated with nano NO• micelles or NO•@PLT.


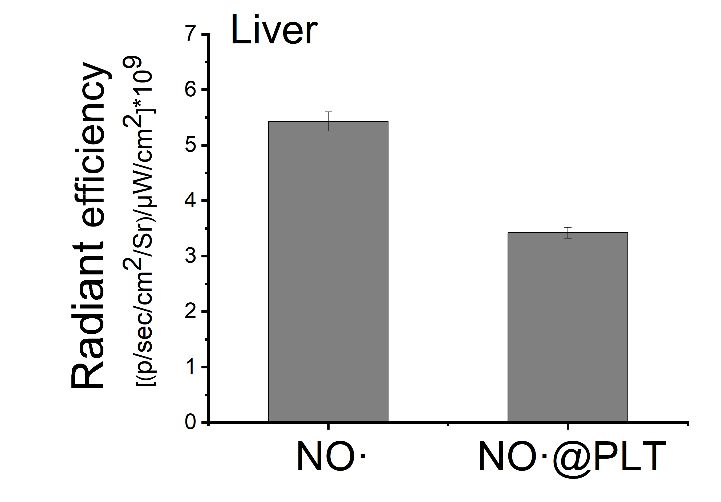


Figure S7. Fluorescence intensity quantification of liver after treated with nano NO• micelles or NO•@PLT.


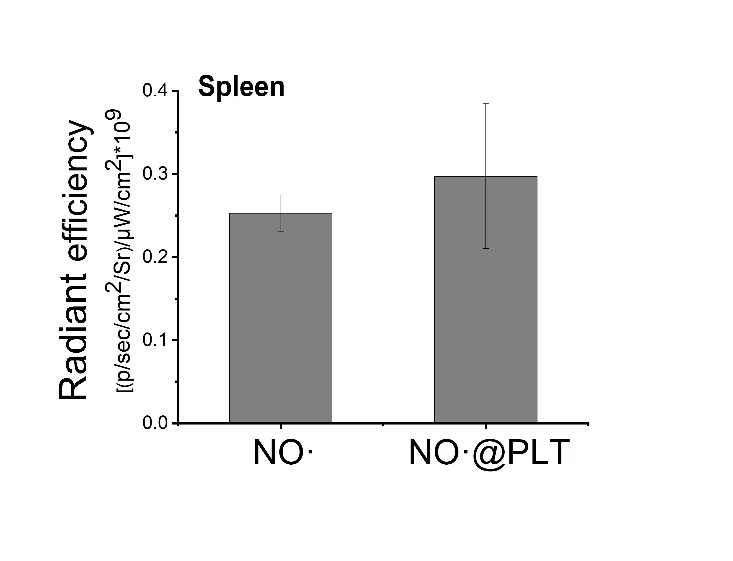


Figure S8. Fluorescence intensity quantification of spleen after treated with nano NO• micelles or NO•@PLT.


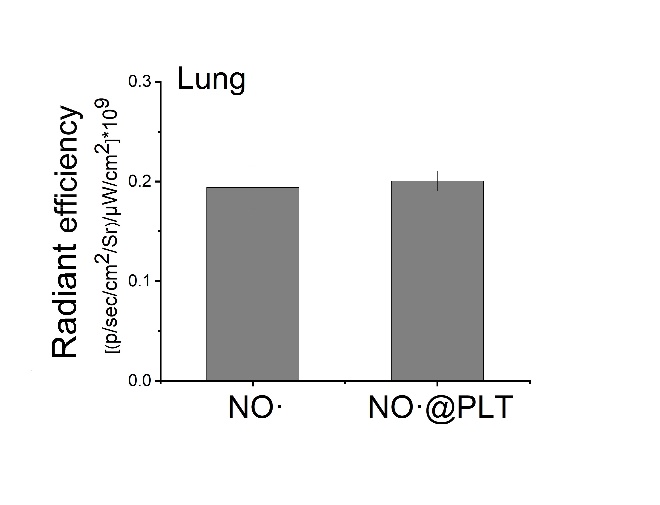


Figure S9. Fluorescence intensity quantification of lung after treated with nano NO• micelles or NO•@PLT.


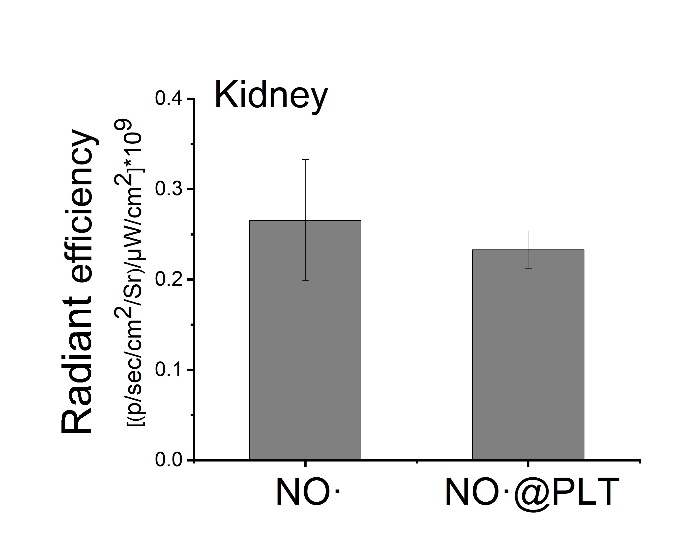


Figure S10. Fluorescence intensity quantification of kidney after treated with nano NO• micelles or NO•@PLT.


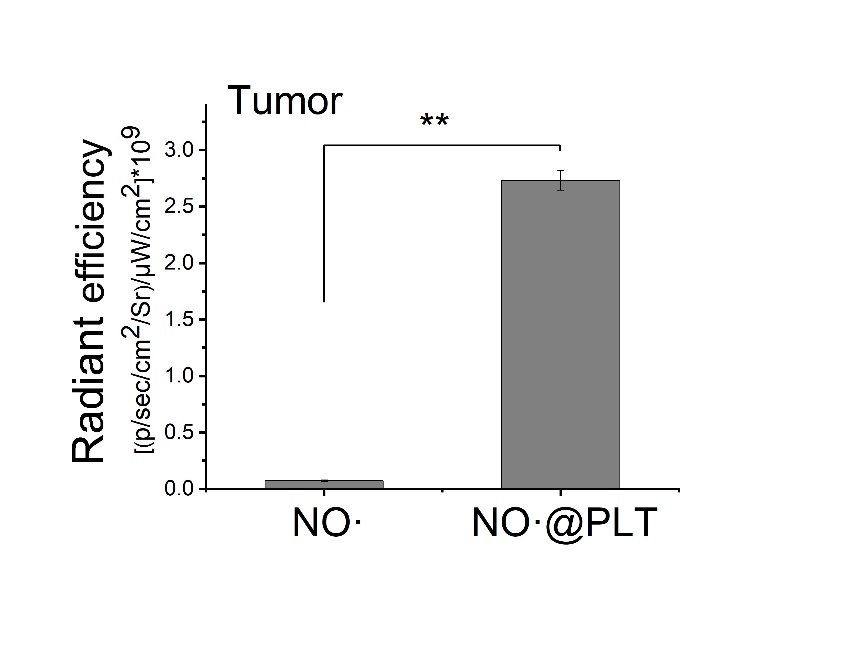


Figure S11. Fluorescence intensity quantification of tumor after treated with nano NO• micelles or NO•@PLT.


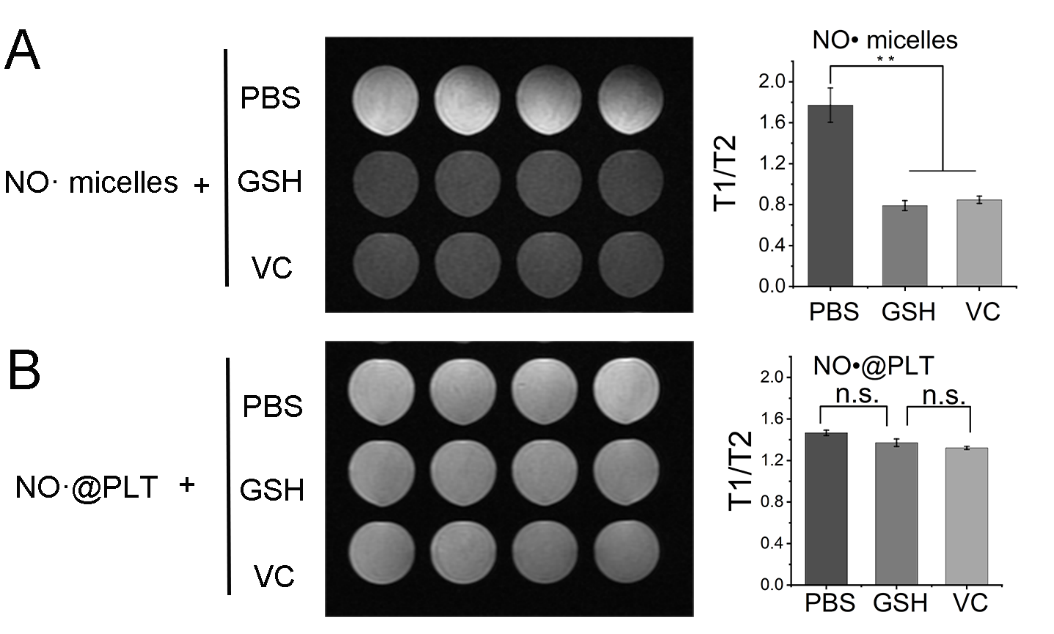


Figure S12. Anti-interference ability test. (A) T1 weighted images of nano NO• micelles after added with 0.1 mL of PBS, glutathione (GSH) or ascorbic acid (VC). The changes in signal intensity as indicated by T1WI/T2WI was also showed. (B) T1 weighted images of NO•@PLT after added with o.1 mL of PBS, glutathione (GSH) or ascorbic acid (VC). The instability comes from reducing substances in body such as glutathione and ascorbic acid. After the instabilities were added, the NO•@PLT showed good anti-interference performance, while there was a significant decrease in the signal intensity (T1WI/T2WI) in the nano NO• micelles group after the GSH or VC was added (** *P*< 0.01). The PLT drug carriers can overcome the poor *in vivo* stability of nitroxide-based MRI contrast agents.


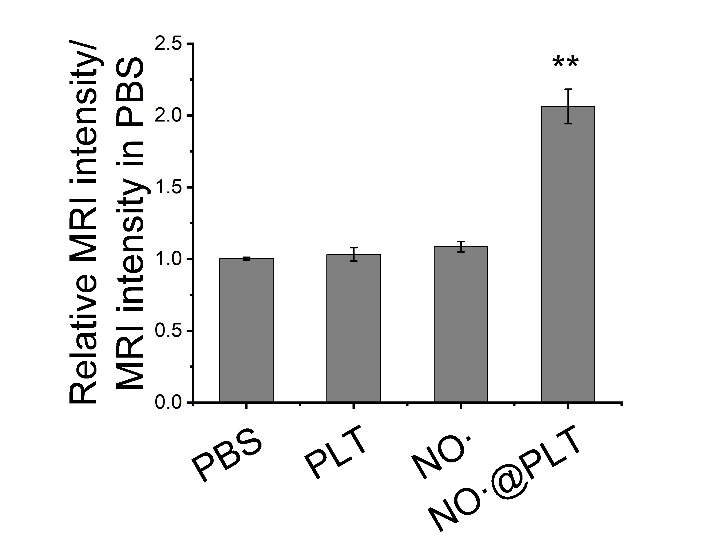


Figure S13. The changes of MRI intensity as indicated by relative MRI intensity/ MRI intensity in PBS treated group of (Figure 6A).


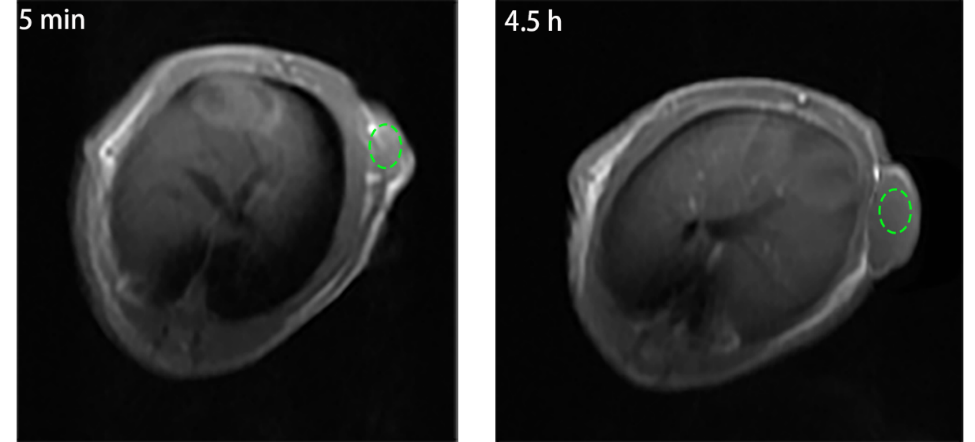


Figure S14. T1-weighted MR images of subcutaneous brain tumor after NO•@PLT administration for 5min and 4.5 h.


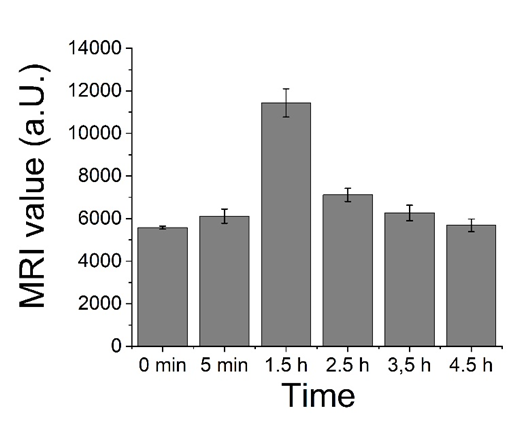


Figure S15. The changes of MRI value after NO•@PLT administration with time.


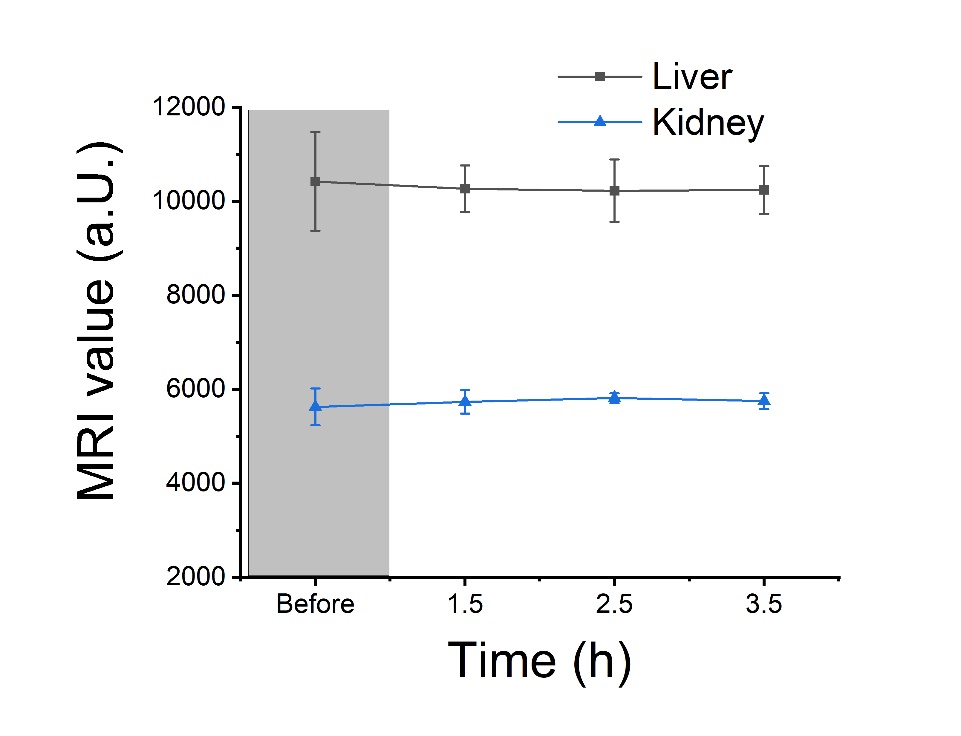


Figure S16. The changes of MRI intensity in liver and kidney after NO•@PLT administration of (Figure 6C).
